# Spatio-temporal overview of neuroinflammation in an experimental mouse stroke model

**DOI:** 10.1038/s41598-018-36598-4

**Published:** 2019-01-24

**Authors:** Lara Buscemi, Melanie Price, Paola Bezzi, Lorenz Hirt

**Affiliations:** 1 Stroke Laboratory, Neurology Service, Department of Clinical Neurosciences, University Hospital Centre and University of Lausanne, CH-1011 Lausanne, Switzerland; 2 Department of Fundamental Neurosciences, University of Lausanne, CH-1005 Lausanne, Switzerland

## Abstract

After ischemic stroke, in the lesion core as well as in the ischemic penumbra, evolution of tissue damage and repair is strongly affected by neuroinflammatory events that involve activation of local specialized glial cells, release of inflammatory mediators, recruiting of systemic cells and vascular remodelling. To take advantage of this intricate response in the quest to devise new protective therapeutic strategies we need a better understanding of the territorial and temporal interplay between stroke-triggered inflammatory and cell death-inducing processes in both parenchymal and vascular brain cells. Our goal is to describe structural rearrangements and functional modifications occurring in glial and vascular cells early after an acute ischemic stroke. Low and high scale mapping of the glial activation on brain sections of mice subjected to 30 minutes middle cerebral artery occlusion (MCAO) was correlated with that of the neuronal cell death, with markers for microvascular changes and with markers for pro-inflammatory (IL-1β) and reparative (TGFβ1) cytokines. Our results illustrate a time-course of the neuroinflammatory response starting at early time-points (1 h) and up to one week after MCAO injury in mice, with an accurate spatial distribution of the observed phenomena.

## Introduction

Stroke is a major public health issue, ranking as second cause of death worldwide and first cause of handicap in adults. It also ranks second, after ischemic heart disease for the estimation of lost years of healthy life^1^. Major improvements have been achieved in the acute phase management of ischemic stroke, firstly by expanding the use of thrombolysis with recombinant tissue-plasminogen activator in the extended 4.5 h time-window^2^. Secondly, a remarkable efficacy of endovascular clot removal within 7.3 h from symptom onset was recently demonstrated^3^. Carefully selected patients were shown to benefit from endovascular treatment up to 24 h after symptom onset^4,5^. Despite this striking progress, many patients die or remain disabled and the effort to understand finely stroke pathophysiology needs to be sustained.

Ischemic stroke is the result of a permanent or transient occlusion of a major brain artery or one of its branches; hence it is a cerebrovascular disease. For decades the main focus of stroke research, and in particular of neuroprotection approaches was set on preventing neuronal loss after ischemic injury. More recently, the trend is shifting towards viewing neuroprotection from a broader point of view, not focusing only on protecting neurons but also addressing the altered physiology of glial cells and of the vascular compartment that can also be involved in delayed damage and brain repair mechanisms^6–8^. The neurovascular unit is an important structural and functional entity, essential for normal brain functioning and homeostasis^9^. Pathological conditions such as cerebral ischemia have a major impact on all elements of the neurovascular unit, namely neurons, astrocytes, juxtavascular microglia, endothelial cells and vascular mural cells. While, typically, neurons are damaged or even die, the other constituents of the neurovascular unit are also affected and surviving cell populations evolve morphologically and functionally to orchestrate a complex chain of events to confine and repair the damage caused by the injury. Here we provide a detailed and visual assessment of the changes that affect neurons, glial and endothelial cells over time after an ischemic stroke in the mouse brain. We used immunofluorescence analysis to identify the different cell types and built maps covering large areas of the mouse brain at different time-points, with striking patterns of tissue response to cerebral ischemia. Although earlier studies have investigated some of these tissue changes, we provide here a comprehensive and visual global assessment of the interconnected modifications. We then further show the evolution of the spatio-temporal changes at higher magnification, focusing on individual cell types. Providing a detailed analysis of the progression of the multicellular response occurring in the neurovascular unit after ischemic stroke is an important step towards a better understanding of its spontaneous healing response that could pave the way for carefully targeted therapeutic approaches. It could also provide a well-characterised model for scientists interested in specific aspects of glial pathophysiology.

## Results

### Overview of the main cellular types involved in neuroinflammation

**Figure 1:**
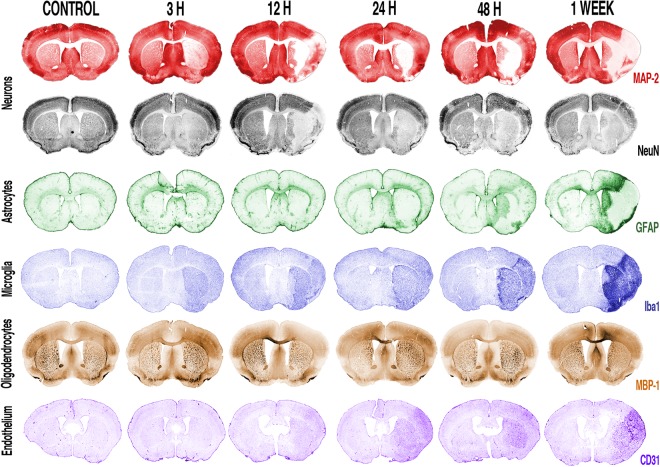
Coronal overview of the spatio-temporal evolution of the glial and vascular reaction to 30 minutes transient middle cerebral artery occlusion with reperfusion. Coronal pseudo-coloured images. Neurons were stained with MAP-2 (red) or NeuN (grey), reactive astrocytes were stained with GFAP (green), microglial cells were stained with Iba1 (blue), oligodendrocytes were stained with MBP-1 (dark yellow) and endothelial cells with CD31 (purple) on coronal sections of mouse brain at the time-points indicated above the images. The images shown are representative of at least three animals per time-point.

**Figure 2:**
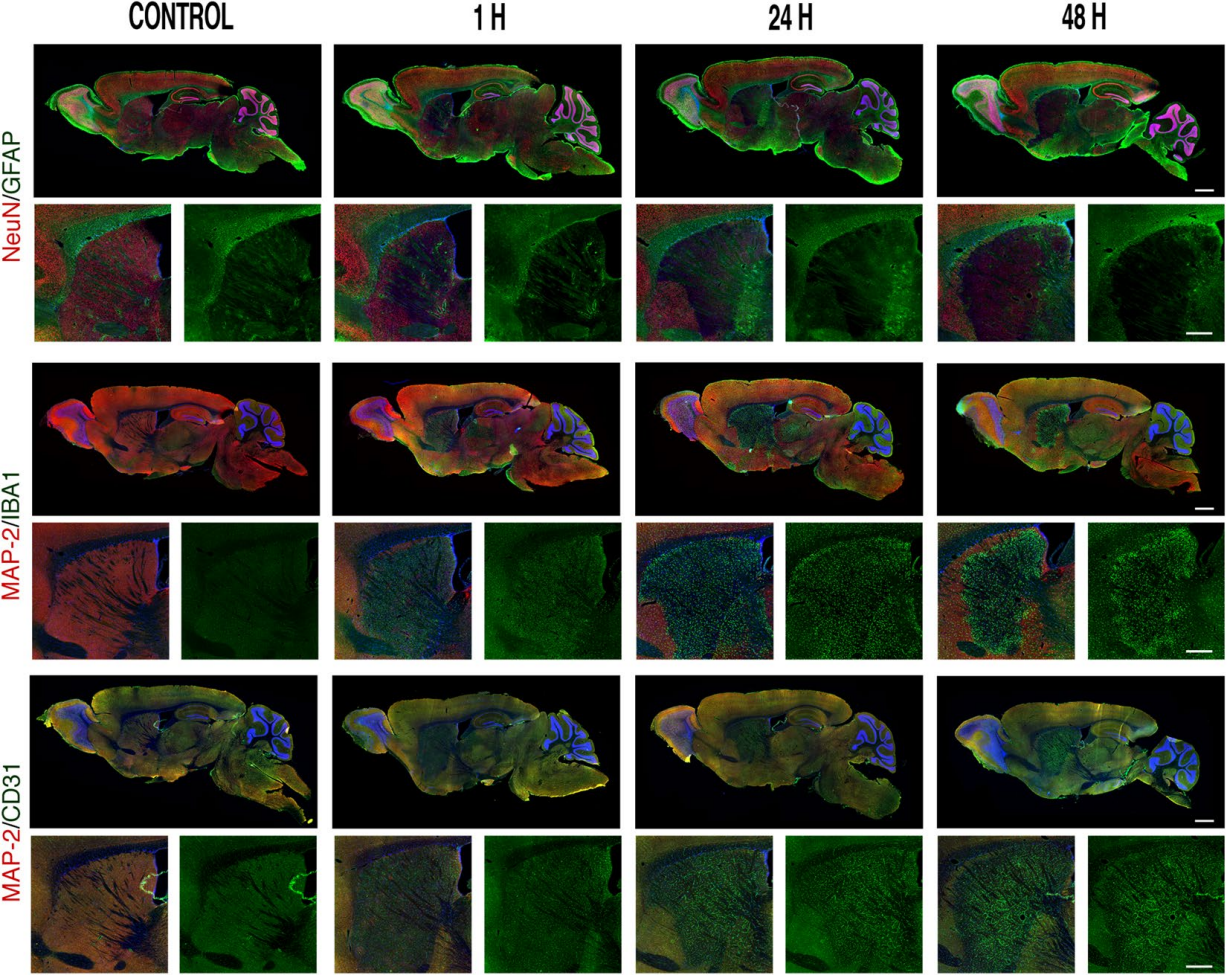
Sagittal overview of the spatio-temporal evolution of the glial and vascular reaction to 30 minutes transient middle cerebral artery occlusion with reperfusion. Double immunostaining showing the spatio-temporal relation of glial (astrocytes, GFAP staining in green; microglia, Iba1 staining in green) and vascular endothelial cells (CD31 staining in green) with respect to the lesion (loss of NeuN or MAP-2 staining in red) on a sagittal view encompassing a broad array of brain areas. Scale bars: 1 mm. Under each double staining sagittal image there is a higher-resolution cropping of the striatal area showing the temporal evolution for astrocytes (GFAP, green), microglia (Iba1, green) and endothelial cells (CD31, green). Scale bars: (500 μm).

An overview of the territorial changes in the main components of the neurovascular unit and their interplay is provided on coronal (Fig. 1) and sagittal (Fig. 2) sections of brains from mice sacrificed at different time-points after experimental stroke. Computerised reconstructions of immunostained sections map the extent of the neuronal damage (loss of MAP-2 or NeuN signal) and its spatial relation with post-stroke glial (GFAP for astrocytes, Iba1 for microglia, MBP-1 for oligodendrocytes) and vascular (CD31 for endothelial cells) reactions.

The mouse striatum is the brain structure most commonly affected by the intraluminal suture MCAO procedure^10^. Hence, coronal sections showing the striatum were immunostained with markers for the neurovascular unit main components at different time-points (Fig. 1). While astrocytes (green sections) slowly become reactive and surround the lesion tissue (loss of staining in upper red sections and grey sections), microglia (blue sections) show a much faster reaction and populate the area displaying neuronal death and higher vascular reactivity (lower purple sections). To note that no evident gross changes are observable in the MBP-1 staining (oligodendrocytes, dark-yellow panels) until one week after the MCAO.

To provide an extended point of view of the interplay between the different cell types and the lesion, we performed immunostaining on sagittal cuts of mouse brains (Fig. 2). The first two rows of images show the staining of neurons (NeuN, red) and of astrocytes (GFAP, green) and confirm what is shown in the coronal cuts, i.e. that the astrocytic reaction evolves at a slowish pace and on the periphery of the lesion, surrounding it. The middle two rows show the double staining of neurons (MAP-2, red) and of microglia (Iba1, green). Note how quickly highly reactive microglia populate the lesion area, which, although patchy, is evident already at one hour after reperfusion and how they show reactivity in areas that do not yet show neuronal loss. The last pair of rows show the double staining of neurons (MAP-2, red) and endothelial cells lining the blood vessels (CD31, green). Note the higher immunoreactivity of the endothelial cells in the damaged area that increases with time, suggesting a neo-vascularization phenomenon, even more prominent at one week (Figs 1, 2).

### Neuronal damage

**Figure 3:**
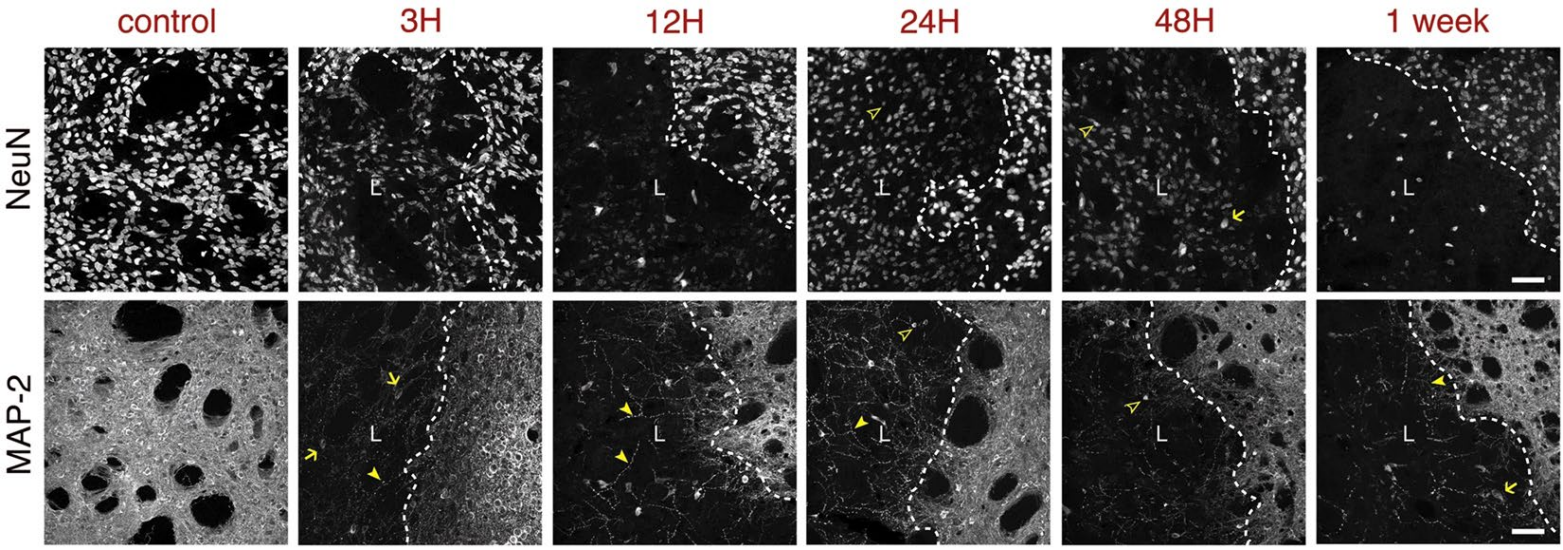
Time-course of neuronal loss after MCAO. (**a**) Neurons were immunostained with NeuN to illustrate the evolution of the lesion in the ipsilateral striata of mice subjected to 30 minutes MCAO at the time-points indicated above the images. (**b**) Similarly, neurons were immunostained with a different neuronal marker, MAP-2. A dashed line indicates the boundary of the lesion (L). Arrows indicate swollen cell bodies; arrowheads indicate neurite beading; hollow arrowheads indicate smaller, condensed nuclei. The images shown are representative of at least three animals per time-point. Scale bars: 50 μm.

Neurons are very sensitive to ischemia. The spatio-temporal evolution of neuronal damage after MCAO is illustrated at low (Figs 1, 2) and high (Fig. 3) magnification using MAP-2 or NeuN staining. We observe the lesion core, especially using MAP-2 as neuronal marker, as early as one hour after reperfusion (Fig. 2), although damage is seen in patches. The lesion is clearly visible and can be already quite extensive by 12 h. However, even though MAP-2 signal is lost rather quickly, observing the NeuN staining at early time-points (up to 24 h) it is possible to see that the signal from neuronal somata, albeit weaker, is still present (Fig. 3). MAP-2 is a cytoskeletal protein strongly expressed in dendrites and less prominent on cell bodies and even though it is likely that at early time-points a degree of MAP-2 loss is attributable to the neuronal death, altered MAP-2 levels could also be an indicator of dendritic stability. Loss of MAP-2 neurite labelling is demonstrated at higher magnification (Fig. 3) at the three hours time-point, which also shows swelling of MAP-2 labelled cell bodies. From 12 h, within the lesion area, the few remaining neurites stained with MAP-2 show beading, a typical morphological modification of neurites after ischemia^11^. In parallel, NeuN immunoreactivity, mostly detected in neuronal cell bodies, becomes weaker from one hour (Fig. 2). At higher magnification and especially at later time-points (e.g. from 24 h) the size of many NeuN labelled nuclei is smaller, which most likely corresponds to pyknosis, reflecting apoptotic neuronal death. Overall, Fig. 3 shows that the neuronal markers NeuN and MAP-2 both provide useful and distinct information.

### Astrocytes

**Figure 4:**
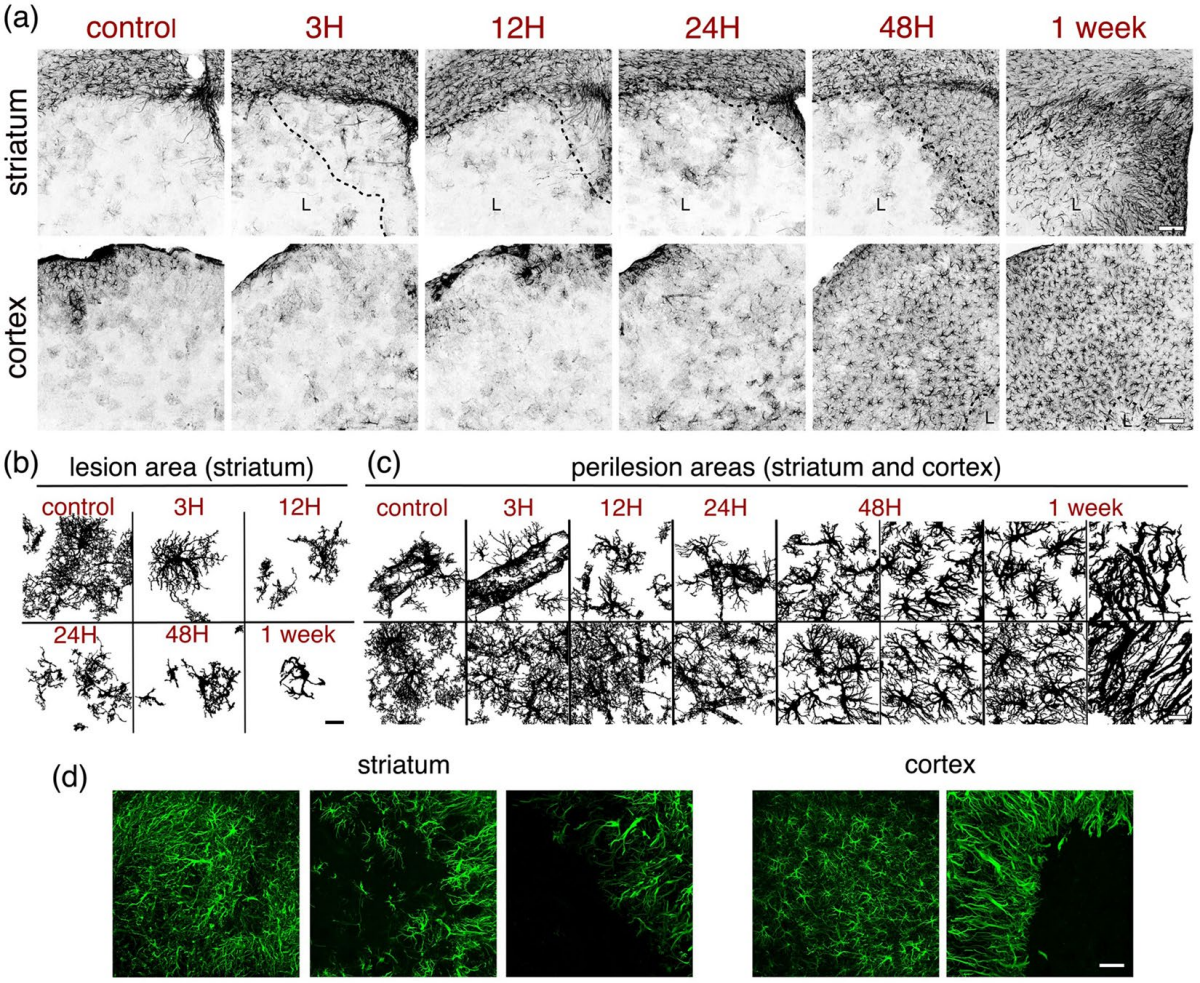
Reactive astrocytes. (**a**) Astrocytes from the ipsilateral striatum (top panels) or cortex (bottom panels) of control mice or mice subjected to 30 minutes MCAO were immunostained with GFAP to illustrate the evolution of the astrocytic reaction at the time-points indicated above the images. A dashed line indicates the boundary of the lesion (L). Scale bar: 100 μm. (**b**,**c**) Morphological changes of typical astrocytes found within the striatal lesion (**b**) or in the peri-lesion areas (**c**) of mice subjected to MCAO are shown using images that were thresholded and subsequently binarized. Scale bars: 20 μm. In 48 h (**c**) and one-week (**c**) images, the two left panels correspond to astrocytes in areas distal from the lesion, whereas the two right panels correspond to astrocytes in proximal areas bordering the lesion. (**d**) Images of the different boundaries between astrocytes (GFAP, green) and damaged tissue taken in striatum (left) and cortex (right). The images shown are representative of at least three animals per time-point. Scale bar: 50 μm.

Reactive astrogliosis is a key component of the cellular response to CNS injury^12^. Astrocytic changes following MCAO are illustrated using an antibody against glial acidic fibrillary protein (GFAP), a marker of reactive astrocytes (Figs 1, 2, 4). In control brains, there is a strong labelling of fibrous astrocytes in the corpus callosum (Fig. 4a “striatum”) and astrocytes in the glia limitans (Fig. 4a “cortex”), together with a weaker labelling of some perivascular astrocytes, which cover the circumference of vessels with their endfeet (Fig. 4c). After ischemia, hypertrophic reactive astrocytes appear at first (24–48 h) and scar-forming astrocytes later (48 h-one week). Although the gradient of astrocytic diversity is not as clear as that observed in trauma-induced injuries^13^, there is topographic diversity of astrocytes with distance from the lesion, as well as locally among neighbouring cells. Whereas the hypertrophic reactive astrocytes are seen in the more external part of the peri-lesion area, closer to healthy tissue, the scar-forming astrocytes are seen at the boundary of the lesion, with very prominent GFAP labelling at one week (Figs 1, 4a). In the lesion core area (i.e. the striatum) at three hours, the astrocyte morphology appears mainly normal, whereas it evolves towards cell body swelling, shortening of the processes and loss of ramification already from 12 h to one week after injury (Fig. 4a top panels “striatum” and 4b). Conversely, in the peri-lesion (Fig. 4a,c), reactive astrocytes do not become prominent until at least 24 h post-ischemia. The most striking changes are observed as larger robustness of the main processes, with loss or retraction of the very fine processes in the hypertrophic astrocytes (see left panels of the four panels for black-and-white 48 h and one-week images, Fig. 4c). In the case of the scar-forming astrocytes bordering the lesion, there is a clear directionality to the extension of the thick processes towards the lesion (see right panels of the four panels for black-and-white 48 h and one-week images, Fig. 4c) and a high degree of process intertwining, especially one week after the injury. Note that there is some variability in the delimitation of the lesion border by astrocytes. In some cases, there is a wall-like alignment of GFAP-positive astrocytes defining a sharp border and in other cases; the border appears to be less sharp with a more progressive distribution of astrocytes and intertwining of their long processes into the lesion (Fig. 4d). Overall, following MCAO there is a large variety of astrocyte morphologies, as illustrated in Fig. 4b and c, depending on the time-point and distance from the lesion.

### Microglia

**Figure 5:**
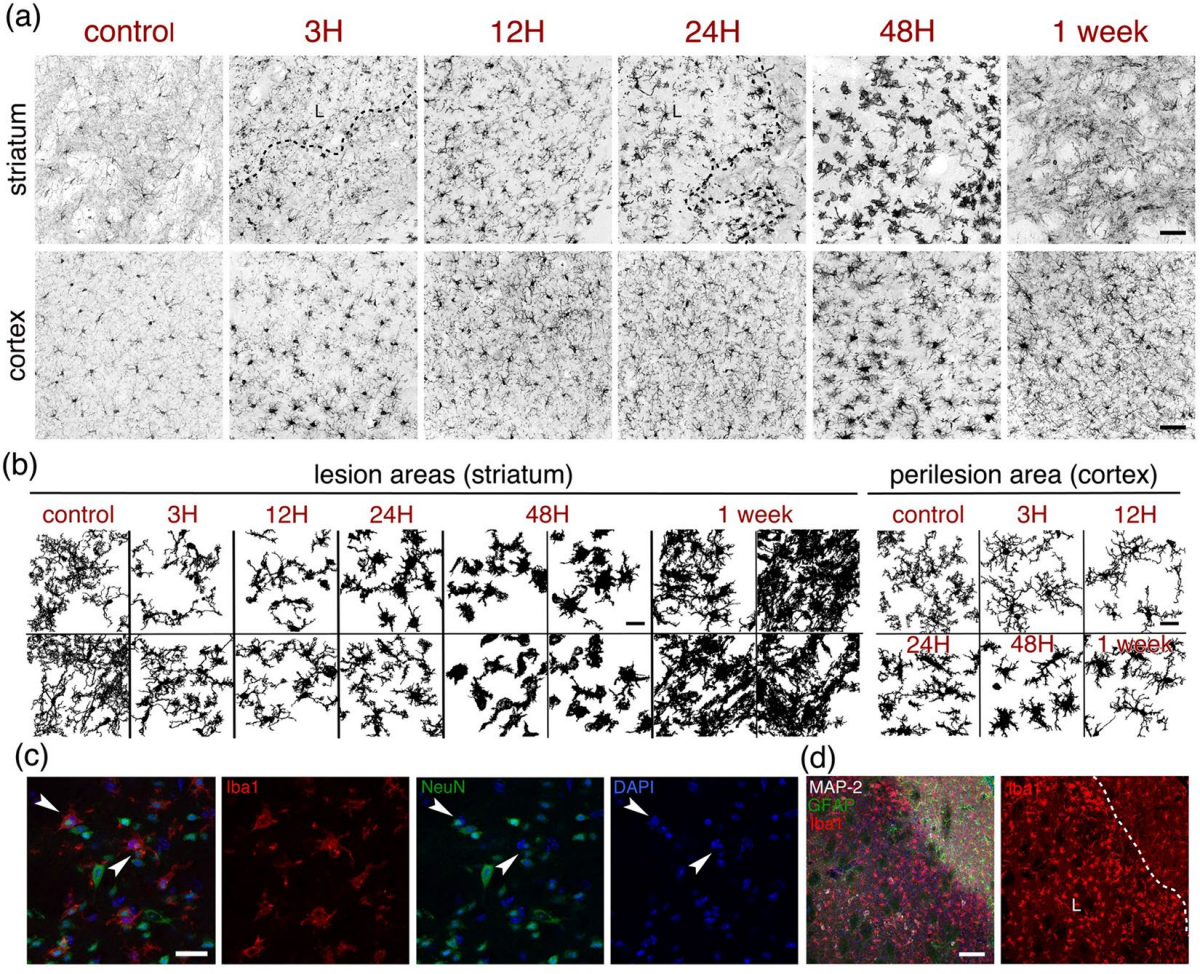
Microglia. (**a**) Microglial cells from the ipsilateral striatum (top panels) or cortex (bottom panels) of control mice or mice subjected to 30 minutes MCAO were immunostained with GFAP to illustrate the evolution of the astrocytic reaction at the time-points indicated above the images. A dashed line indicates the boundary of the lesion (L). Scale bars: 50 μm. (**b**) Morphological changes of typical microglial cells found within the striatal lesion (left) or in the peri-lesion areas (right) of mice subjected to MCAO are shown using images that were thresholded and subsequently binarized. Scale bar: 20 μm. (**c**) Single plane images of microglial cells stained with Iba1 (red), neurons stained with NeuN (green) and nuclear counterstaining (DAPI, blue) showing engulfment of neurons with condensed nuclei (arrowheads) by microglial cells, 48 h after MCAO onset. Scale bar: 25 μm. (**d**) Image of the boundary between the penumbral area and the lesion core, defined by the presence or absence of MAP-2 neuronal staining (grey) and delimited by the astrocytic barrier (GFAP staining, green), showing the morphological differences of microglial cells (Iba1 staining, red) on either side of the barrier, with DAPI as nuclear counterstaining, 48 h after MCAO onset. A dashed line indicates the boundary of the lesion (L). The images shown are representative of at least three animals per time-point. Scale bar: 50 μm.

We labelled microglia using an antibody against Iba1 (ionized calcium-binding adapter molecule 1; Figs 1, 2, 5). Since infiltration of peripheral macrophages into the brain is low for the first few days after MCAO^14^, Iba1-positive cells are likely to be mainly microglia. Iba1 immunoreactivity increases from one hour, within the lesion, gradually up to one week, where the intralesional Iba1 labelling is very strong (Figs 1, 2). At higher magnification (Fig. 5) one sees at three hours, the long, thin and highly ramified microglial processes observed in healthy controls retract. Microglia have been shown to respond to loss of signalling from injured neurons^15^ and indeed, microglia in the infarct core seem to be initially injured as a result of the ischemia. Processes remain retracted at 12 h. At 24 h, microglia become hypertrophic, with enlarged cell bodies, and extend long, wide processes while by 48 h, they become more rounded, amoeboid-like, still extending processes that engulf nearby structures, especially abundant in the inner boundary of the ischemic lesion. Interestingly, we show phagocytosis of NeuN-labelled neurons by activated microglia at 48 h (Fig. 5d). At one week after ischemia, increased numbers of Iba1-labelled immune cells populate the lesion core. However, their shape is more elongated than at 48 h and remarkably, their distribution respects the architecture of the striatum, as cells seem to clearly avoid the white matter bundles that are anatomically characteristic of this brain structure (Fig. 5c). Although the most prominent microglial activation occurs within the lesion, there are also changes in Iba1-reactivity and microglial morphology in the peri-lesional cortex (Fig. 5a,c). The major changes in microglial morphology in the peri-lesion seem to happen at 48 h post-injury, although at one-week after ischemia; strong activation in penumbra is still evident. Two days after reperfusion, in the boundary zone between the ischemic lesion core and the penumbral area populated by abundant GFAP positive cells, (Fig. 5d) amoeboid cells in the ischemic core can be distinguished from highly ramified microglia on the other side of the astrocytic barrier. Engulfment of stressed but viable neurons (which may expose phosphor-serine) in the ischemic penumbra of experimental stroke lesions amplifies overall tissue damage and worsens behavioural outcomes^16,17^. Hence, the astrocytic scar, acting as a barrier to the penetration of reactive microglia or even inflammatory cells infiltrated from the periphery, would be a protective entity.

### Proinflammatory and repair-promoting cytokines

**Figure 6:**
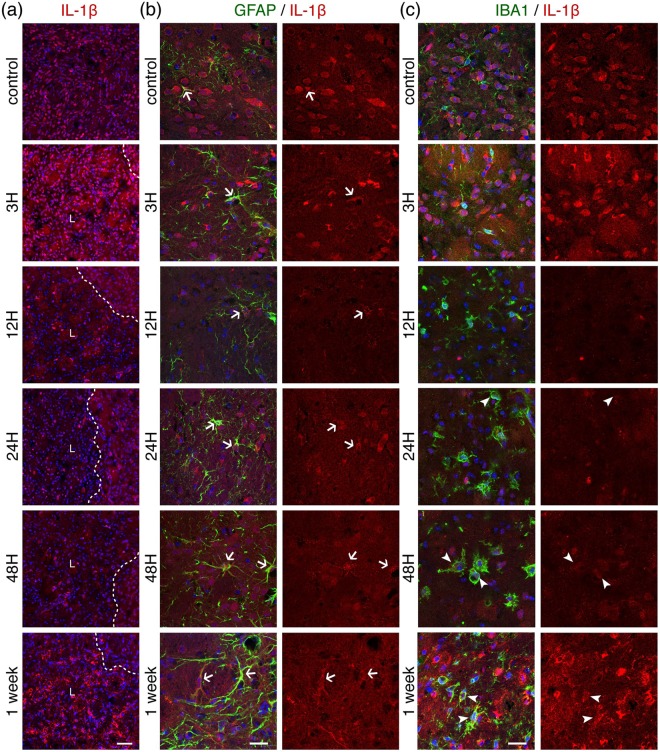
Temporal evolution of the proinflammatory marker Interleukin-1β (IL-1β) after 30 minutes MCAO. (**a**) Low magnification images from ipsilateral to lesion striatal tissue sections taken at different time-points after ischemia and immunostained for IL-1β (red). A dashed line indicates the boundary of the lesion (L). Scale bar: 50 μm. Sections were counterstained for reactive astrocytes ((**b**), GFAP staining in green) or microglia ((**c**), Iba1 staining in green) and shown at higher magnification. Arrows (astrocytes) or arrohweads (microglia) point towards cells that express IL-1β. DAPI (blue) was used as a nuclear counterstaining. The images shown are representative of at least three animals per time-point. Scale bars: 20 μm.

**Figure 7:**
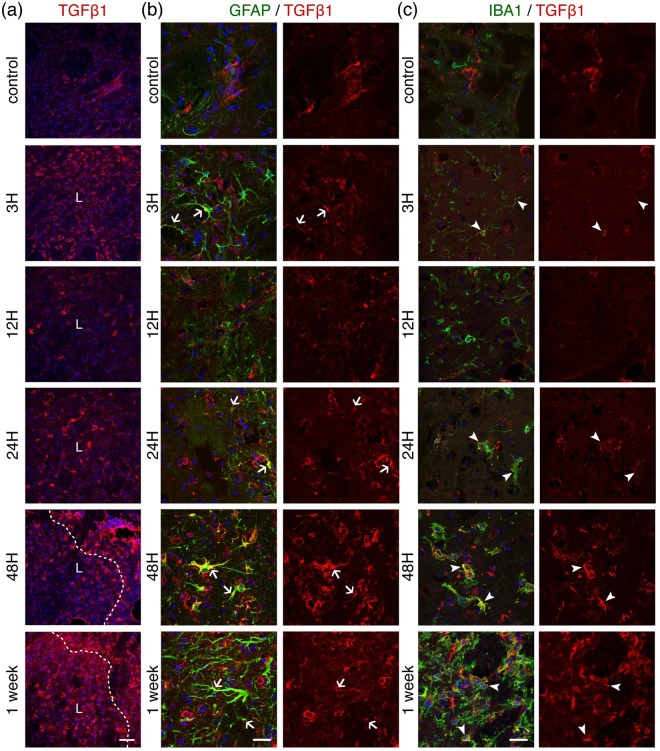
Temporal evolution of the tissue-repair cytokine Transforming Growth Factor β1 (TGFβ1) after 30 minutes MCAO. (**a**) Low magnification images from ipsilateral to lesion striatal tissue sections taken at different time-points after ischemia and immunostained for TGFβ1 (red). A dashed line indicates the lesion boundary (L). Scale bar: 50 μm. Sections were counterstained for reactive astrocytes ((**b**), GFAP staining in green) or microglia ((**c**), Iba1 staining in green) and shown at higher magnification. Arrows (astrocytes) or arrohweads (microglia) point towards cells that express TGFβ1. DAPI (blue) was used as a nuclear counterstaining. The images shown are representative of at least three animals per time-point. Scale bars: 20 μm.

**Figure 8:**
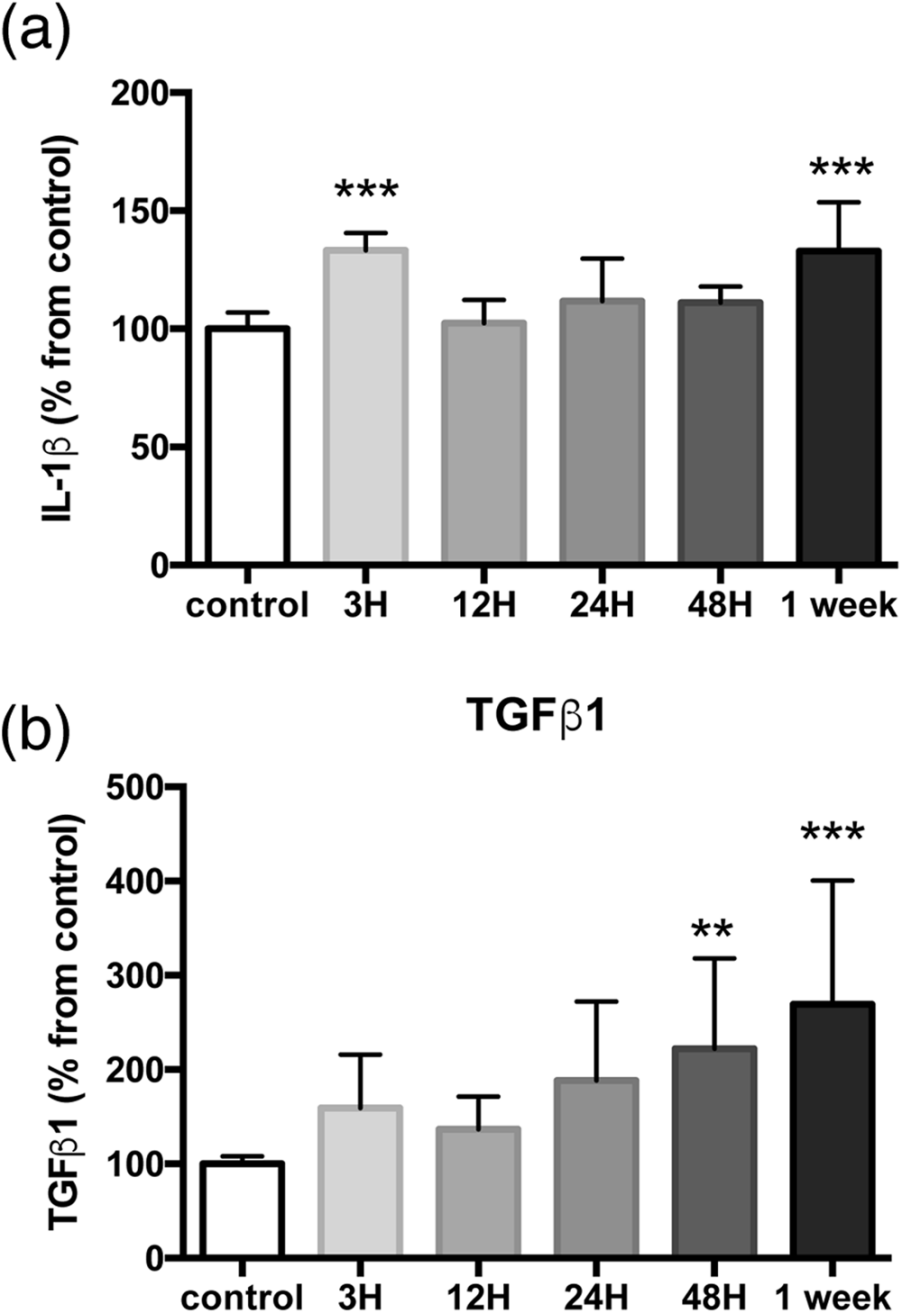
Semi-quantitative evaluation of IL-1β and TGFβ1. Temporal evolution of tissue IL-1β (**a**) and TGFβ1 (**b**) levels, normalized to controls. The graphs show the mean and standard deviation of images collected on samples from two (IL-1β) or three (TGFβ1) different sets of experiments. **p < 0.01; ***p < 0.001, one-way ANOVA with Tukey *post-hoc* test.

We looked at the expression of the proinflammatory cytokine IL-1β (Fig. 6) and the repair-promoting cytokine TGFβ1 (Fig. 7). IL-1β is detected in the striatum of control mice. After MCAO, there are two waves of IL-1β expression, a large widespread diffuse increase at three hours and a more focalized one in what looks like localized accumulation of the cytokine at one week (Fig. 6a, Fig. 8a). Double-labelling experiments show coexpression of IL-1β with both GFAP (Fig. 6b) and Iba1 (Fig. 6c). The cytokine is detected in GFAP-positive cells in healthy controls and shows a gradual increase after ischemia, with a punctate labelling pattern in the cell body and along astrocytic main processes (as seen in single-plane confocal images, Fig. 6b). Note that the images of GFAP positive cells in the higher magnification panels from 24 h onwards are in the peri-lesion and not all cells are positive for IL-1β. On the other hand, microglial cells do not appear to substantially express IL-1β until 24–48 h after MCAO (Fig. 6c). Especially one week after ischemia, we observe different degrees of IL-1β expression in Iba1-positive cells present in the lesion core.

The repair-promoting cytokine TGFβ1 is weakly expressed in the striatum in control conditions (Fig. 7). After MCAO, similarly to IL-1β, its expression increases in two waves, slightly at three hours and more pronouncedly at 48 hours-one week (Figs 7a, 8b). Double-labelling experiments show that TGFβ1 colocalises with both GFAP (Fig. 7b) and Iba1 (Fig. 7c) indicating that both activated astrocytes and microglia express TGFβ1. To note that the images of GFAP-positive cells in the higher magnification panels from 24 h onwards are from the peri-lesion. Interestingly, the expression of TGFβ1 in astrocytes seems stronger at 48 h than one week after ischemia and at the latter time-point; there is abundant TGFβ1 labelling beyond astrocytic and microglial signals. Information on the circulating levels of these two cytokines in the MCAO stroke model can be found elsewhere^18,19^.

### Oligodendrocytes

**Figure 9:**
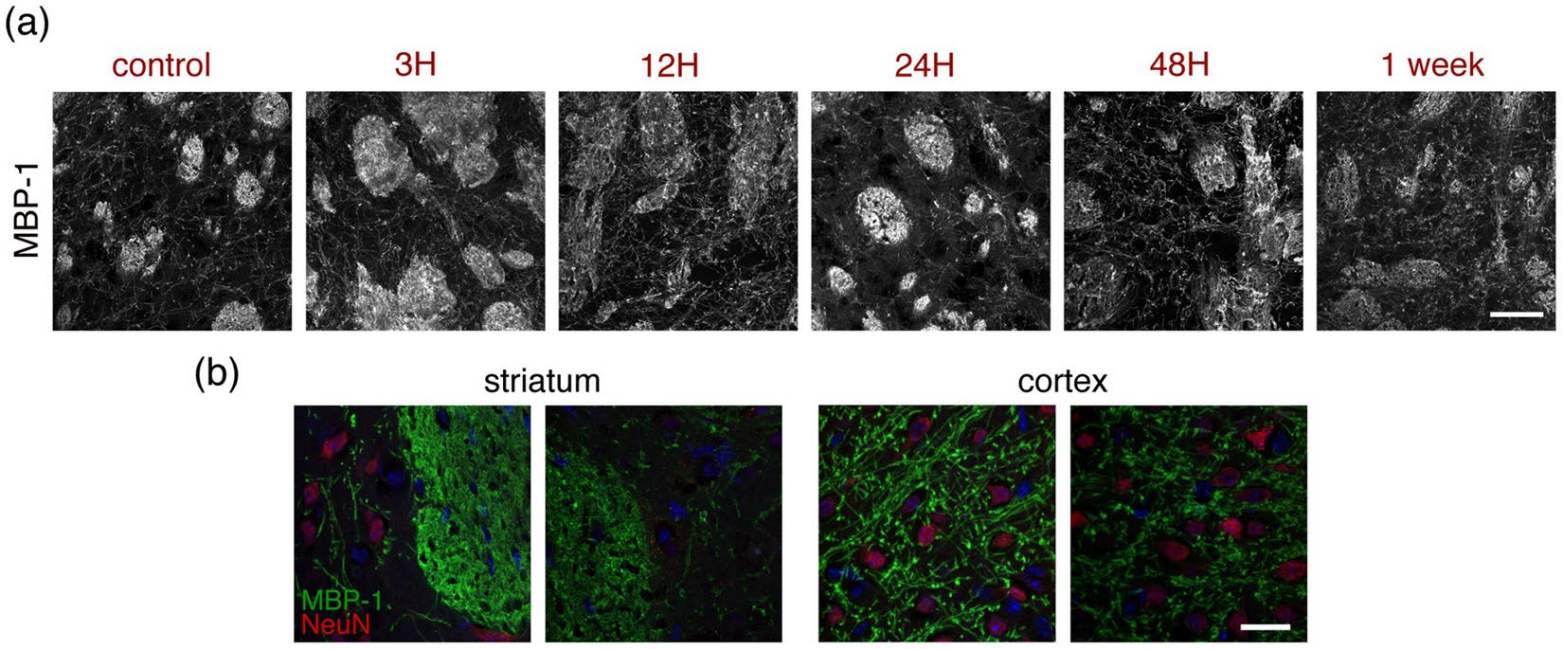
Oligodendrocytes. (**a**) Oligodendrocytes from the ipsilateral striatum of control mice or mice subjected to 30 minutes MCAO were immunostained for Myelin Basic Protein 1 (MBP-1) to illustrate the damage caused to oligodendrocytes at the time-points indicated above the images. Images were taken in the lesion area. Scale bar: 50 μm. (**b**) Higher magnification, single plane images comparing healthy (left panels) and one-week after stroke (right panels) tissue from striatum or cortex stained for MBP-1 (green) and NeuN (neurons, red) with blue DAPI counterstaining. The images shown are representative of at least two animals per time-point. Scale bar: 20 μm.

We labelled oligodendrocytes using an antibody against MBP-1 (myelin basic protein 1). At low magnification (Fig. 1) no striking changes were observed. Changes not evident at low magnification are detectable at higher magnification and mostly seen as a destructuring of the cellular network, first in the lesion area (3 to 24 h) followed by cell loss at later time-points (Fig. 9). At one week, double labelling with NeuN shows reduced MBP-1 immunoreactivity in the peri-lesional cortex and loss of labelling in both processes and cell bodies in the lesion core (striatum).

### Blood vessels

**Figure 10:**
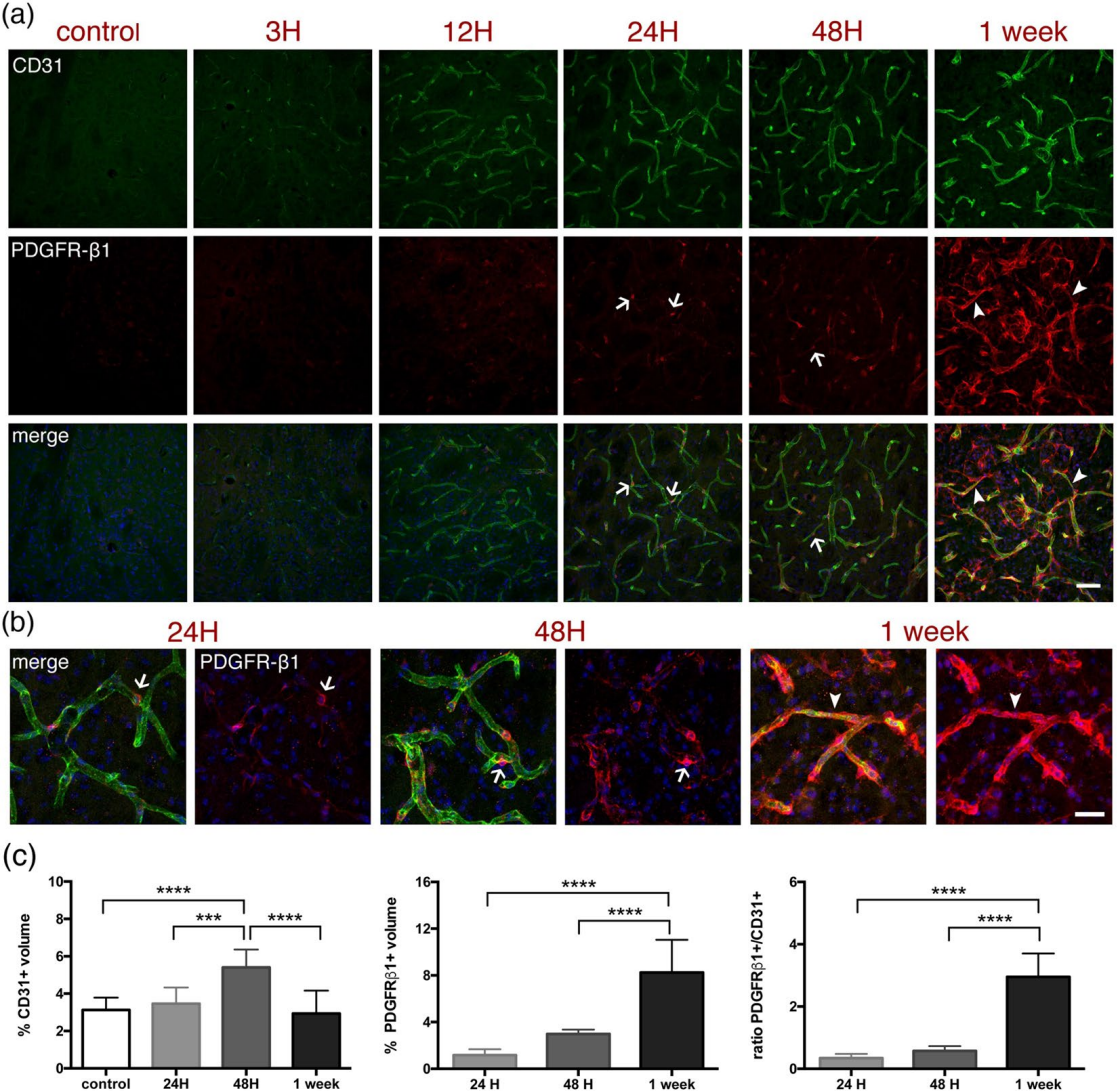
Time-course of the vascular remodelling and stromal reaction after MCAO. (**a**) Endothelial cells (CD31 staining, green) and pericytes and stromal cells (PDGFRβ-1, red) from within the ipsilateral striatum of control mice or mice subjected to 30 minutes MCAO were immunostained to illustrate the evolution of the changes in the vascular system in the lesion as well as the stromal cell reaction at the time-points indicated above the images. Scale bar: 50 μm. (**b**) Higher magnification images taken at selected time-points and immunostained for CD31 (green) and PDGFRβ-1 (red). DAPI (blue) was used as nuclear counterstaining. Arrows indicate pericytes; arrowheads indicate constriction. Scale bar: 25 μm. (**c**) Quantification of the volume of CD31-positive cells (left), PDGFRβ-1-positive cells (middle) and the PDGFRβ-1/CD31 volume ratio (right). To avoid interference of the background noise, only images of PDGFRβ-1 from 24 h onwards were considered. The graphs show the mean and standard deviation of images collected on samples from three different sets of experiments. ***p < 0.001; ****p < 0.0001, one-way ANOVA with Tukey *post-hoc* test. The images shown are representative of at least three animals per time-point. Although the volume of CD31-labelled cells has returned to volumes observed in control mice one week after MCAO, the CD31-labelling intensity remains stronger.

Blood vessels and vascular mural cells were labelled using antibodies against the mainly endothelial protein, CD31 and a pericyte marker, PDGFR-β1. As shown in Figs 1 and 2, there is a strong increase over time of CD31 labelling within the striatal lesion. At higher magnification (Fig. 10a), the same enhancement of CD31 labelling is observed, starting at three hours and becoming very strong at 48 h and one week. The pericyte labelling starts to be quite evident from 12 h and becomes very strong at one week. Interestingly, on double labelling (Fig. 10b), we see that PDGFR-β1-positive cells display a typically ‘bump-on-a-log’ labelling at 24 h (arrows). However, it seems that they completely cover and constrict the capillaries by one week (arrowheads), hinting at an active role in blood-flow restriction at this time-point. In fact, whereas the volume occupied by CD31-positive cells in the lesion increases by almost two-fold at 48 h to decrease to control levels by one week, the volume of PDGFR-β1-positive cells increases several fold at one week (Fig. 10c).

## Discussion

Here we provide a comprehensive and visual mapping of the evolution of the multicellular response to stroke in one of the most widely used mouse models of transient cerebral ischemia. The use of large extension maps allows a better illustration and localization of the territorial boundaries of the bulk post-stroke changes as done classically for lesion extension evaluation. Additionally, the approach of first looking at the ‘big picture’ followed by higher-magnification images to get a closer look at the cellular and molecular events occurring in specific areas of interest gives us a wider perspective of where things happen, an important issue often neglected in a largely inhomogeneous organ like the brain.

Using this strategy we visually illustrate the spatio-temporal evolution of events that fall into each one of the three overlapping but distinct phases of the multicellular response to acute focal CNS damage (such as the one modelled by MCAO) described by Burda and Sofroniew^20^. These are (1) cell death and inflammation, (2) cell proliferation for tissue replacement, and (3) tissue remodelling. During the initial phase of cell death and inflammation, corresponding to our 1–12 h time-points, we observe suffering/loss of neurons, astrocytes and even microglia in the lesion core area (Figs 1–4). These events are temporally correlated with an overall increase of the proinflammatory cytokine IL-1β (Fig. 6, first wave of increase at three hours). The 24–48 h time-points correspond to cell proliferation and tissue replacement phase. At these time-points we observe increased numbers of GFAP-positive cells in the peri-lesion area (Fig. 4) and an increase in vascular reactivity within the striatal lesion core that goes together with a notable presence of peri-vascular PDGFRβ-1-positive cells (Fig. 9), suggestive of neo-vascular remodelling. In addition, the activity of phagocytic microglia clearing debris associated with neuronal death (Fig. 5) and the strong signal for TGFβ1 in astrocytes, microglia and other cells in the lesion (Fig. 7) favour the idea of tissue modification-replacement. Finally, in the third phase of tissue-remodelling (48h-one week), there is massive presence of PDGFRβ-1-positive cells, not only covering the vessels but also in the parenchyma of the lesion, indicative of stromal cells, hence of a fibrotic scar. Also the establishment of a clear astrocytic scar delimiting the vast invasion of inflammatory cells into the neighbouring healthy tissue, concomitant with a large expression of TGFβ1 and IL-1β in the tissue devoid of neurons. Both the damaged and remaining healthy tissue will progress into this third phase, but the main aim of our time-course was to describe the early days after infarction.

Traditional preclinical stroke research has aimed therapy design at preventing neuronal death, immediate or delayed. However, besides the immediate neuronal damage caused by the harsh environmental conditions due to ischemia, a considerable amount of the post-stroke damage to CNS tissue is via secondary injury, through microglial activation, capillary pericyte constriction and numerous other mechanisms. Interestingly, this early potentially deleterious response to acute injury is also the trigger for the reparative events that follow. It is of note that therapeutic interventions targeting post-ischemic inflammation should be mindful of temporal considerations, minimizing the destructive potential of inflammation in the acute phase while enhancing its beneficial contributions to tissue repair in the late stages of cerebral ischemia^8^. Indeed, learning how, when and where these protective mechanisms are triggered and implemented may be of great help in the quest to treat stroke. Recently, therapies targeted to non-neuronal cell populations have aimed to abrogate indirect neuronal degeneration, as well as to enhance endogenous repair mechanisms^21^. Interestingly, the most successful trial for endovascular stroke therapy to date^4^ was based on choosing patients that had salvageable penumbral tissue, tissue that is clearly affected by the neuroinflammation and repair processes we are imaging at low scale. Furthermore, it has been recently shown that some benefits of reperfusion by endovascular clot removal may go beyond the rescue of neurons by enhancing the brain’s repair mechanisms^22^.

Neuroprotection strategies targeting the functional integrity of astrocytes, for example, may constitute a superior strategy for future neuroprotective therapies. It has been proposed that the astrocyte-scar border serves as a protective barrier that restricts the migration of inflammatory cells from the injured lesion core into surrounding viable neural tissue^13,23^. These functional astrocytic barriers sharply demarcate tissue compartments in which robust inflammatory responses essential for clearing debris can develop^13^ and set a permissive environment for the creation of a dense fibrotic scar made by stromal cells^24^, while preserving immediately adjacent viable and functional neural tissue. This compartmentalization can be observed after experimental stroke as illustrated by the differences in microglial morphology on either side of the astrocytic barrier in Fig. 5d, with the amoeboid-like cells being those that phagocyte condensed neuronal nuclei confined within the lesion (Fig. 5c,d), and importantly it can also be observed after stroke in humans^25^. In cerebral ischemia, mutant mice lacking an efficient astroglial scar in the case of a vimentin and GFAP double knockout^26^ or in the absence of Caveolin-1^27^ develop larger lesions and impaired neurological outcome. The astroglial scar not only keeps phagocytic reactive microglia at bay, but also neatly delimitates the fibrotic scar as cellular and molecular constituents of the fibrotic scar induce the repulsion and polarization of astrocytes^6^. Furthermore, both scars seem to act in a cooperative way, as it has been suggested that efficient fibrotic formation within areas of infarct may promote peri-infarct astrogliosis^28^. A better understanding of the multicellular and molecular interactions that determine the location of astrocyte scar borders around areas of compromised tissue may lead to novel therapeutic strategies for reducing lesion size. These events occur over a time frame of days after the insult, offering a favorable therapeutic window during which intervention is a realistic possibility.

Another interesting feature of the brain recovering from stroke that could be considered as a target for therapy is the complex pattern of vascular remodeling. Angiogenesis is promoted by hypoxia^29^ and it has been detected in rodent models of cerebral ischemia^30^. We show a striking change in vascular labelling with enhanced immunoreactivity for the endothelial marker CD31 within the striatal ischemic lesion (Figs 1,2). With a slight delay we observe the increased expression of the pericyte and stromal cell marker PDGFR-β1 and an apparent widening of the vessels at 24–48 h post-injury (Fig. 9). As metabolic and clearance needs of the brain tissue might drive the delivery of blood flow^31^, this widening could be related to a potential need of cerebral blood flow increase in order to clear the brain of potentially toxic by-products of brain activity, as well as for brain thermoregulation. Contrarily, at one week our images show constriction of capillaries by PDGFR-β1 positive cells, suggesting a role in cerebral blood flow control. Also, pericytes are necessary for endothelial cells in the blood-brain barrier to repress endothelial transcytosis^32^. Further experiments are required to correlate our observations with blood-flow regulation and blood-brain barrier function.

A typical way of evaluating the efficacy of a treatment in preclinical stroke research has been to assess the reduction of neuronal death extension after the intervention as a primary outcome measure. However, it is becoming evident that the functional outcome is not always necessarily correlated with the overall extension of the lesion core^33^. It is beyond the scope of this work to relate a particular astrocyte, microglial or vascular morphology or distribution to a better functional outcome. However, it will be interesting to measure the effects of a therapeutic intervention both in terms of lesion size and the associated multicellular response that accompanies the different recovery phases post-stroke. Similarly, it would be interesting to correlate the distribution of the astrocytic scar and the microglial response with the behavioural outcome of mice after stroke following therapeutic interventions and to find what kind of intervention might promote better establishment of an astrocytic isolating-scar without further compromising the viability of the tissue. One potential approach to find new therapies could be to take into account the beneficial aspects of the repair processes that happen naturally after the damage and potentially enhance them for a better or faster functional recovery, thus lowering the disability index associated with stroke.

## Materials and Methods

### Animal Model

All experiments were performed in accordance with Swiss Laws for the protection of animals and were approved by the Vaud Cantonal Veterinary Office. Male C57BL/6J mice (37 animals, body weight 19–25 g, aged 4–8 weeks; Charles River, France) had free access to food and water and were maintained on a 12 h light-dark cycle in a temperature- and humidity-controlled animal facility. Surgeries were performed under isoflurane anesthesia (1.5–2% in 70% N_2_O/30% O_2_) using a facemask. Mice were administered buprenorphine (0.025 mg/kg, subcutaneous injection) at the beginning of the surgery for post-surgery analgesia. Rectal temperature was monitored throughout surgery and maintained at 37.0 ± 0.5 °C using a heating pad (FHC Inc., USA). A flexible probe was fixed on the skull at 1 mm posterior and 6 mm lateral from bregma to measure and record throughout the surgery regional cerebral blood flow (rCBF) by laser-Doppler flowmetry (Perimed AB, Sweden). Transient focal cerebral ischemia was induced by introducing through the left common carotid artery into the internal left carotid artery a silicon-coated nylon suture (Doccol Corp, USA) to occlude the left middle cerebral artery (MCA) for 30 minutes, after which the occluding filament was withdrawn to allow reperfusion, as described previously^34,35^. Surgery was considered successful if rCBF was <20% of baseline during the 30 minutes occlusion and >50% of baseline after reperfusion. After the surgery mice were housed in an incubator at 28° C until sacrifice or overnight.

At indicated time-points after ischemia-reperfusion, three (3 h, 12 h) to six (control, 24 h, 48 h, 1 week) mice per time-point for the coronal sections and one mouse per time-point for the sagittal sections were sacrificed by intraperitoneal injection of 150 mg/kg sodium pentobarbital and intracardially perfused with 4% paraformaldehyde in phosphate buffer at pH 7.4.

### Immunostaining

For immunofluorescence labelling, 40 μm free-floating cryostat sections (25 μm sections for the sagittal cuts) were pre-incubated in 10% horse serum and 0.3% Triton X-100-PBS-1% BSA and then incubated over two days at 4 °C with primary antibodies in 2% horse serum and 0.3% Triton X-100-PBS-1% BSA (0.1% Triton X-100 and overnight incubation for the 25 μm sections), washed in PBS, and incubated in Alexa Fluor-coupled secondary antibodies (ThermoFisher-Molecular Probes) and DAPI for nuclear counterstaining one hour at room temperature, rinsed in PBS and mounted in FluorSave™ (Calbiochem, #345-789-20). The antibodies used were as follows: anti-Microtubule Associated protein 2 (MAP-2) mouse monoclonal (Millipore #MAB3418, 1:400), anti-Neuronal Nuclear antigen (NeuN) mouse monoclonal (Millipore #MAB377, 1:400), anti-Glial Fibrillary Acidic protein (GFAP) mouse monoclonal (Millipore #MAB3402, 1:2000), anti-GFAP rabbit polyclonal (Millipore #AB5804, 1:2000), anti-Ionized calcium Binding Adaptor molecule 1 (Iba1) goat polyclonal (Abcam #ab5076, 1:400), anti-Platelet Endothelial Cell Adhesion Molecule-1 (PECAM-1, also known as CD31) rat monoclonal (BD Pharmingen #550274, 1:100), anti-Myelin Basic Protein 1 (MBP-1) rat monoclonal antibody (Millipore #MAB386, 1:600), anti-IL-1β antibody (Santa Cruz Biotechnology #sc-7884, 1:100), anti-TGFβ1 mouse monoclonal antibody (R&D Systems #MAB240, 1:200) and anti-platelet-derived growth factor receptor β-1 (PDGFRβ-1) rabbit polyclonal antibody (Santa Cruz Biotechnology #sc-432, 1:200).

### Image acquisition and processing

Images of coronal cuts were taken at 5x magnification with an Axioplan2 microscope (Carl Zeiss, Germany) and manually stitched using ImageJ stitching plugin^36^. Images of sagittal cuts were taken at 10x magnification and stitched with a Zeiss Z1 slide scanner (Carl Zeiss, Germany). Confocal laser microscopy was performed with a TCS SP5 RS – DM6000 confocal scanning microscope (Leica, Germany) at 20x (HC PL Fluotar, NA 0.5, air), 40x (HCX PL APO, NA 1.25, oil) or 63x (HCX APO, NA 1.40, oil) magnification. Unless otherwise stated, representative confocal images are displayed as maximal z-projections. For double or triple labelling, immunofluorescence signals were sequentially acquired in the same section. For the semi-quantitative evaluation of tissue cytokine levels, the raw integrated density of maximal z-projections of images taken at 63x in three random areas of the lesion of two (IL-1β) or three (TGFβ1) mice per time-point was measured and normalized against control samples. For the evaluation of the volumes of CD31- and PDGFRβ-1-positive cells, 63x z-stacks (11–14 images, z = 1.5 μm) of at least three random areas of the lesion of three mice per time-point were smoothed (3D Gaussian filter) and processed with the 3D object counter plugin from ImageJ. The results are expressed as a percentage of the total volume of the z-stack.

Images were processed and quantified with Fiji/ImageJ software (NIH, USA) and Adobe Photoshop CS5 (Adobe, USA).

### Statistical analysis

Comparisons between variables were analyzed by one-way ANOVA followed by Tukey’s *post-hoc* test. All statistical tests were carried on GraphPad Prism 6.0 (GraphPad Software, USA). Significance was considered at p < 0.05.

## References

[1] Feigin VL, Norrving B, Mensah GA (2017). Global Burden of Stroke. Circulation research.

[2] Ahmed N (2010). Implementation and outcome of thrombolysis with alteplase 3-4.5 h after an acute stroke: an updated analysis from SITS-ISTR. The Lancet. Neurology.

[3] Saver JL (2016). Time to Treatment With Endovascular Thrombectomy and Outcomes From Ischemic Stroke: A Meta-analysis. Jama.

[4] Albers, G. W. *et al*. Thrombectomy for Stroke at 6 to 16 Hours with Selection by Perfusion Imaging. **378**, 708–718, 10.1056/NEJMoa1713973 (2018). 10.1056/NEJMoa1713973 PMC6590673 29364767

[5] Nogueira RG (2017). Thrombectomy 6 to 24 Hours after Stroke with a Mismatch between Deficit and Infarct. New England Journal of Medicine.

[6] Fernandez-Klett F, Priller J (2014). The fibrotic scar in neurological disorders. Brain pathology (Zurich, Switzerland).

[7] Iadecola, C. & Anrather, J. Stroke research at a crossroad: asking the brain for directions. *Nature neuroscience* **14**, 1363–1368, 10.1016/j.neuron.2010.07.002 10.1038/nn.2953 (2011). 10.1038/nn.2953 PMC3633153 22030546

[8] Moskowitz MA, Lo EH, Iadecola C (2010). The science of stroke: mechanisms in search of treatments. Neuron.

[9] Iadecola C (2017). The Neurovascular Unit Coming of Age: A Journey through Neurovascular Coupling in Health and Disease. Neuron.

[10] Endres M (1998). Attenuation of delayed neuronal death after mild focal ischemia in mice by inhibition of the caspase family. Journal of cerebral blood flow and metabolism: official journal of the International Society of Cerebral Blood Flow and Metabolism.

[11] Murphy TH, Li P, Betts K, Liu R (2008). Two-photon imaging of stroke onset *in vivo* reveals that NMDA-receptor independent ischemic depolarization is the major cause of rapid reversible damage to dendrites and spines. The Journal of neuroscience: the official journal of the Society for Neuroscience.

[12] Pekny M, Nilsson M (2005). Astrocyte activation and reactive gliosis. Glia.

[13] Wanner IB (2013). Glial scar borders are formed by newly proliferated, elongated astrocytes that interact to corral inflammatory and fibrotic cells via STAT3-dependent mechanisms after spinal cord injury. The Journal of neuroscience: the official journal of the Society for Neuroscience.

[14] Schilling M (2003). Microglial activation precedes and predominates over macrophage infiltration in transient focal cerebral ischemia: a study in green fluorescent protein transgenic bone marrow chimeric mice. Experimental neurology.

[15] Ransohoff RM, Cardona AE (2010). The myeloid cells of the central nervous system parenchyma. Nature.

[16] Brown GC, Neher JJ (2012). Eaten alive! Cell death by primary phagocytosis: ‘phagoptosis’. Trends in biochemical sciences.

[17] Neher JJ (2013). Phagocytosis executes delayed neuronal death after focal brain ischemia. Proceedings of the National Academy of Sciences of the United States of America.

[18] Chapman KZ (2009). A Rapid and Transient Peripheral Inflammatory Response Precedes Brain Inflammation after Experimental Stroke. Journal of Cerebral Blood Flow & Metabolism.

[19] Lambertsen KL, Biber K, Finsen B (2012). Inflammatory Cytokines in Experimental and Human Stroke. Journal of Cerebral Blood Flow & Metabolism.

[20] Burda JE, Sofroniew MV (2014). Reactive gliosis and the multicellular response to CNS damage and disease. Neuron.

[21] Zhao L-R, Willing A (2018). Enhancing endogenous capacity to repair a stroke-damaged brain: An evolving field for stroke research. Progress in Neurobiology.

[22] Tachibana M (2017). Early Reperfusion After Brain Ischemia Has Beneficial Effects Beyond Rescuing Neurons. Stroke.

[23] Sofroniew MV (2015). Astrocyte barriers to neurotoxic inflammation. Nature reviews. Neuroscience.

[24] Schreiber J, Schachner M, Schumacher U, Lorke DE (2013). Extracellular matrix alterations, accelerated leukocyte infiltration and enhanced axonal sprouting after spinal cord hemisection in tenascin-C-deficient mice. Acta histochemica.

[25] Huang L (2014). Glial scar formation occurs in the human brain after ischemic stroke. International journal of medical sciences.

[26] Li L (2008). Protective role of reactive astrocytes in brain ischemia. Journal of cerebral blood flow and metabolism: official journal of the International Society of Cerebral Blood Flow and Metabolism.

[27] Blochet, C. *et al*. Involvement of caveolin-1 in neurovascular unit remodeling after stroke: Effects on neovascularization and astrogliosis. 271678x18806893, 10.1177/0271678x18806893 (2018). 10.1177/0271678X18806893 PMC6928561 30354902

[28] Shen J (2012). PDGFR-beta as a positive regulator of tissue repair in a mouse model of focal cerebral ischemia. Journal of cerebral blood flow and metabolism: official journal of the International Society of Cerebral Blood Flow and Metabolism.

[29] Pugh CW, Ratcliffe PJ (2003). Regulation of angiogenesis by hypoxia: role of the HIF system. Nature Medicine.

[30] Wei L, Erinjeri JP, Rovainen CM, Woolsey TA (2001). Collateral growth and angiogenesis around cortical stroke. Stroke.

[31] Pulsinelli William A, Levy David E, Duffy Thomas E (1982). Regional cerebral blood flow and glucose metabolism following transient forebrain ischemia. Annals of Neurology.

[32] Daneman R, Zhou L, Kebede AA, Barres BA (2010). Pericytes are required for blood-brain barrier integrity during embryogenesis. Nature.

[33] Weber R (2008). Early prediction of functional recovery after experimental stroke: functional magnetic resonance imaging, electrophysiology, and behavioral testing in rats. The Journal of neuroscience: the official journal of the Society for Neuroscience.

[34] Castillo X (2015). A probable dual mode of action for both L- and D-lactate neuroprotection in cerebral ischemia. Journal of cerebral blood flow and metabolism: official journal of the International Society of Cerebral Blood Flow and Metabolism.

[35] Longa EZ, Weinstein PR, Carlson S, Cummins R (1989). Reversible middle cerebral artery occlusion without craniectomy in rats. Stroke.

[36] Preibisch S, Saalfeld S, Tomancak P (2009). Globally optimal stitching of tiled 3D microscopic image acquisitions. Bioinformatics (Oxford, England).

